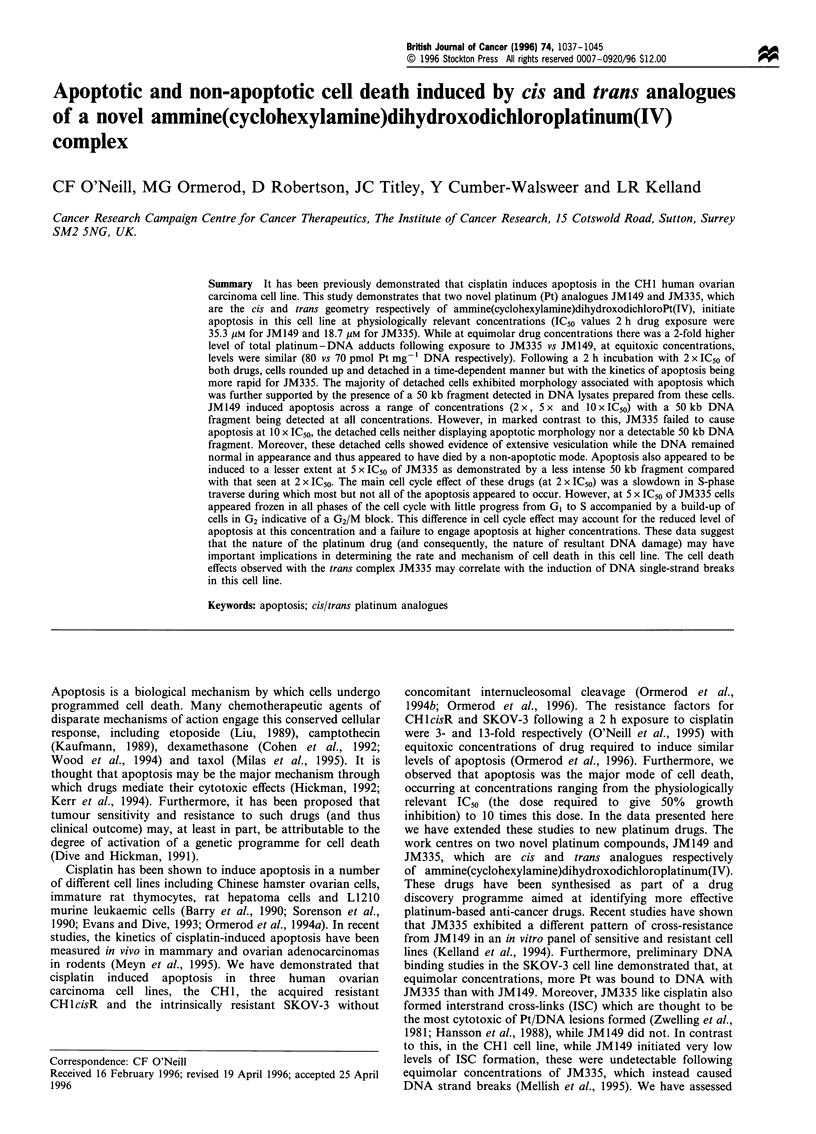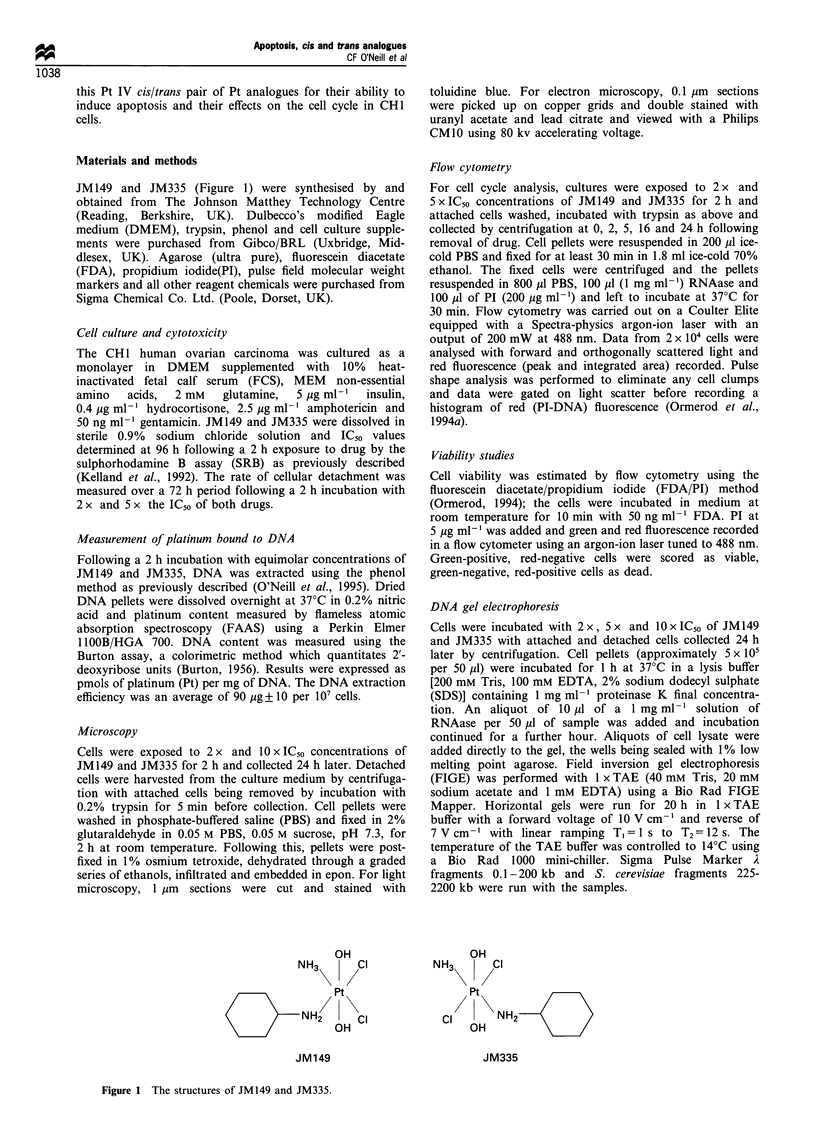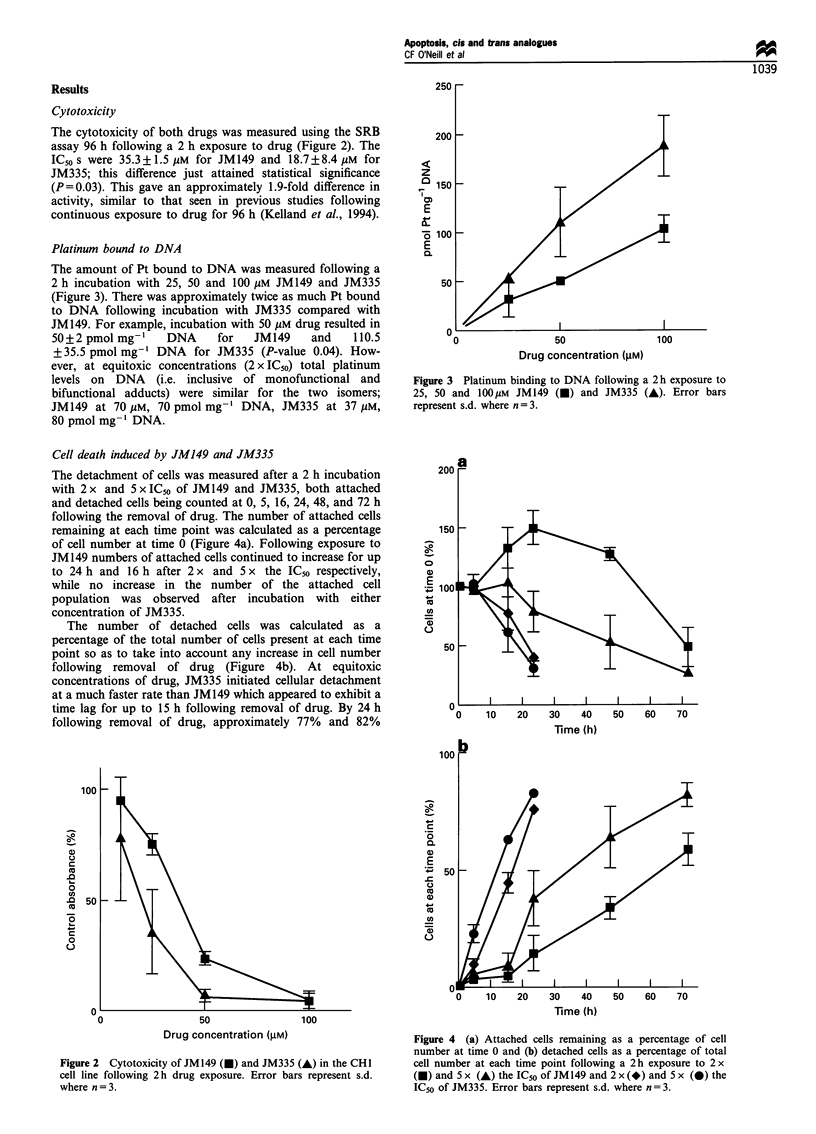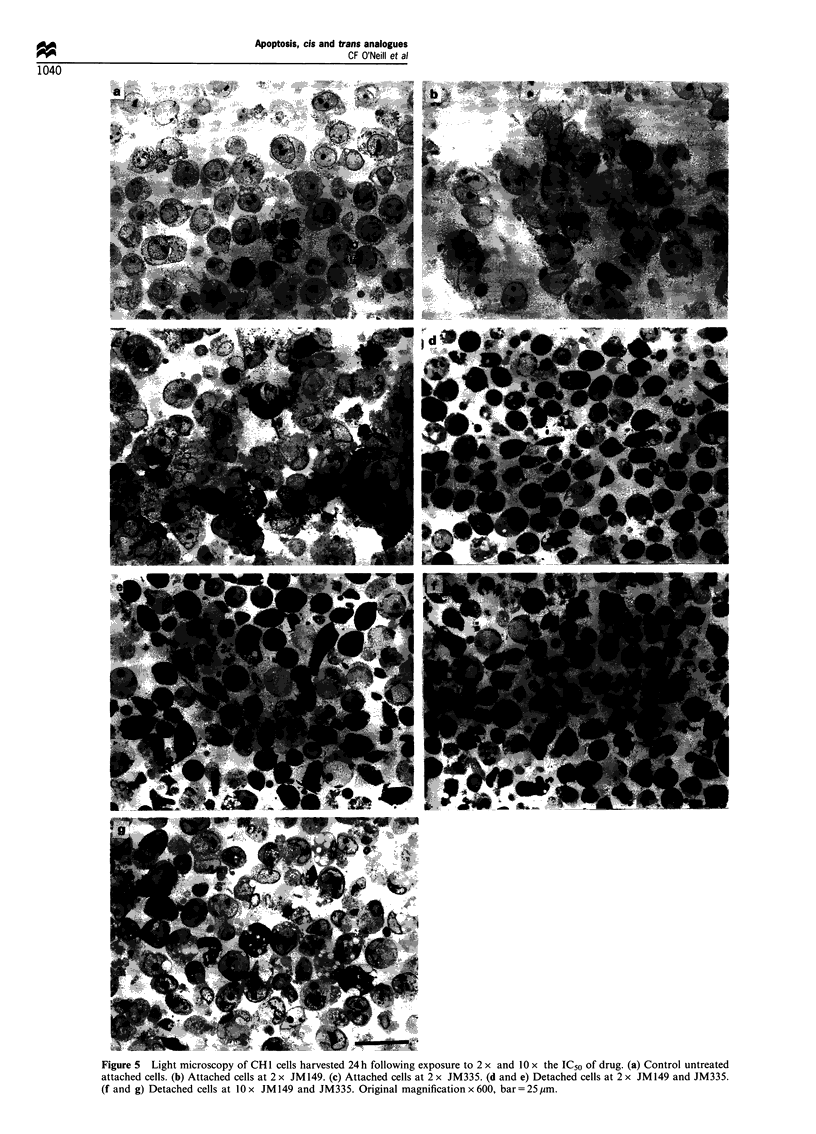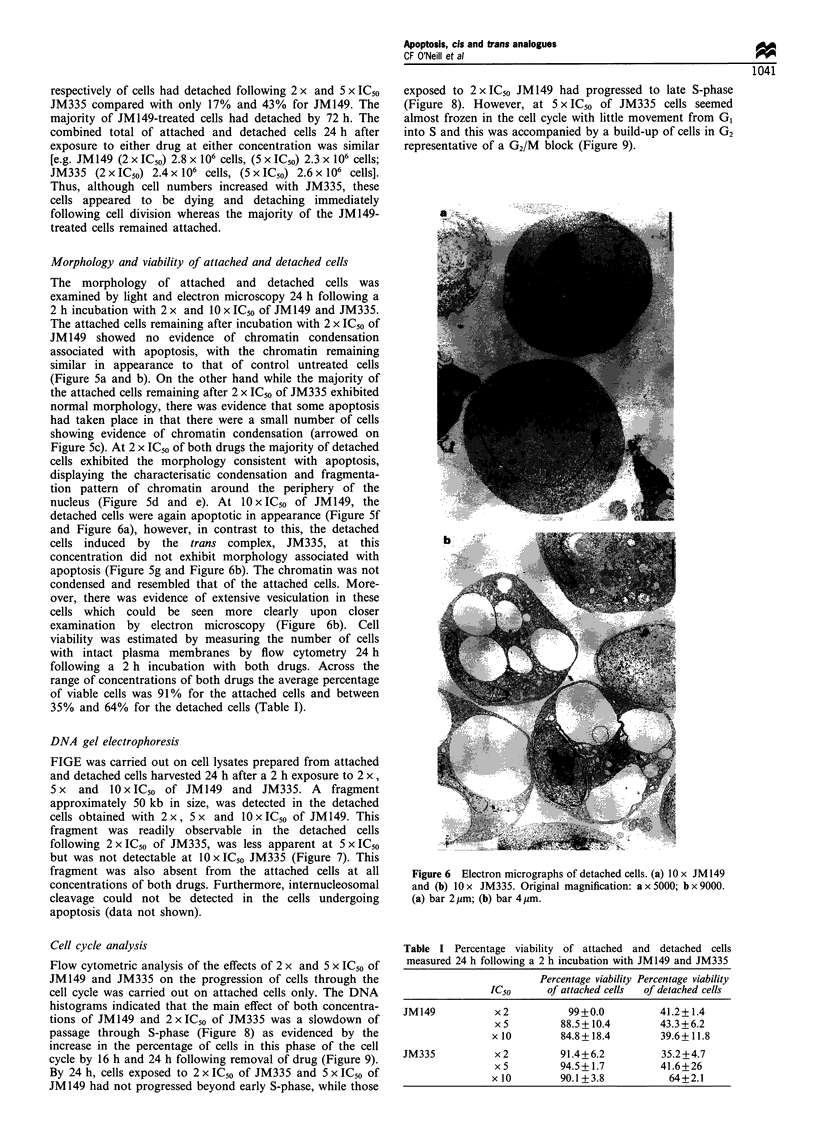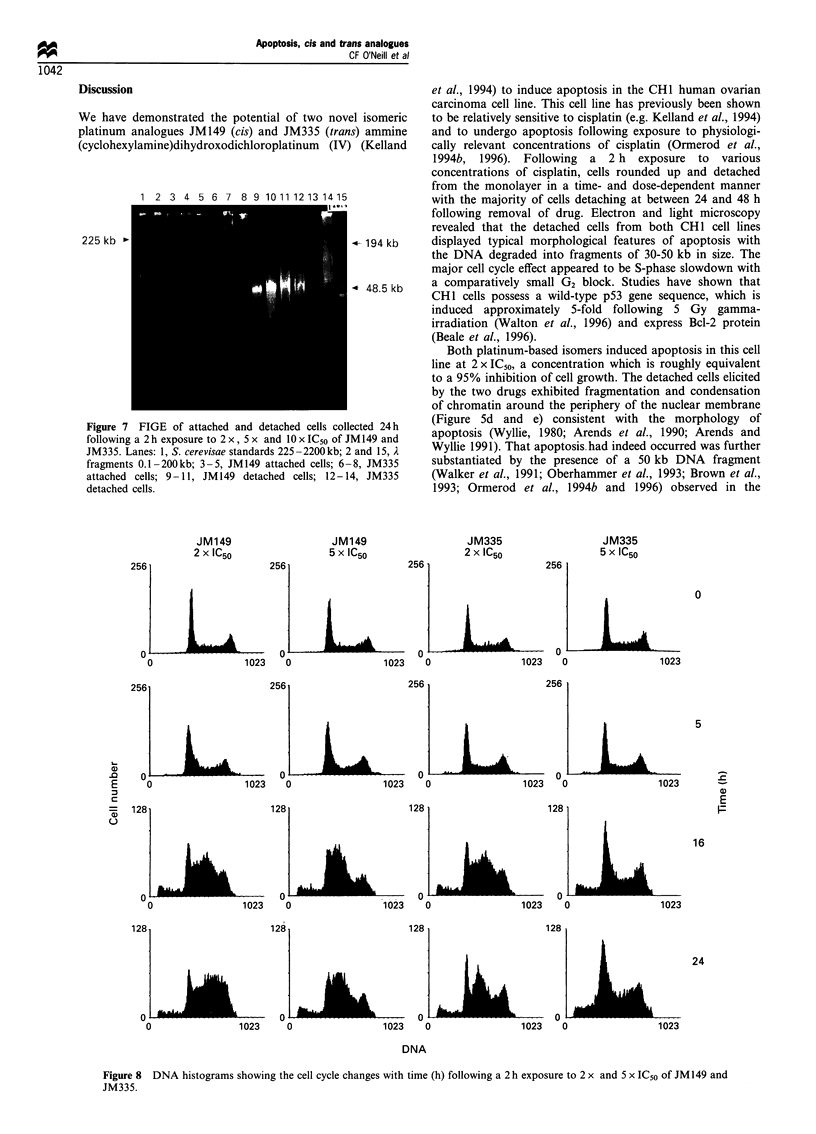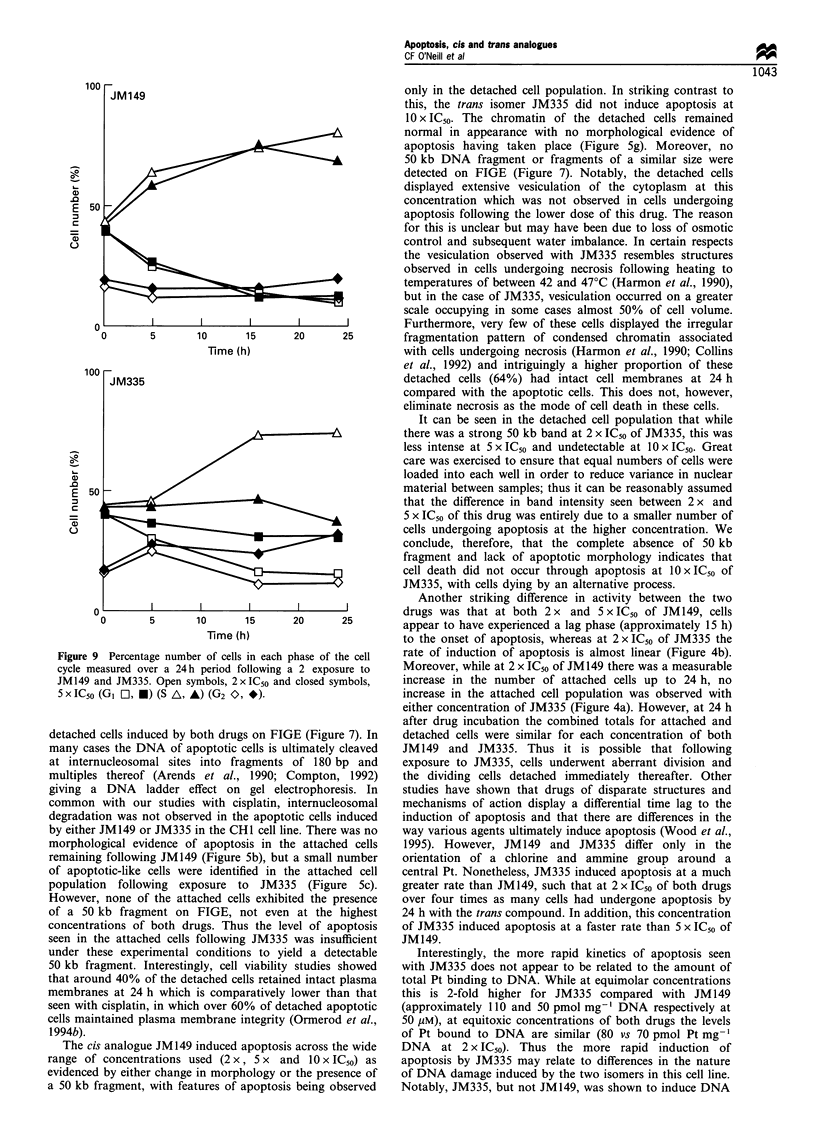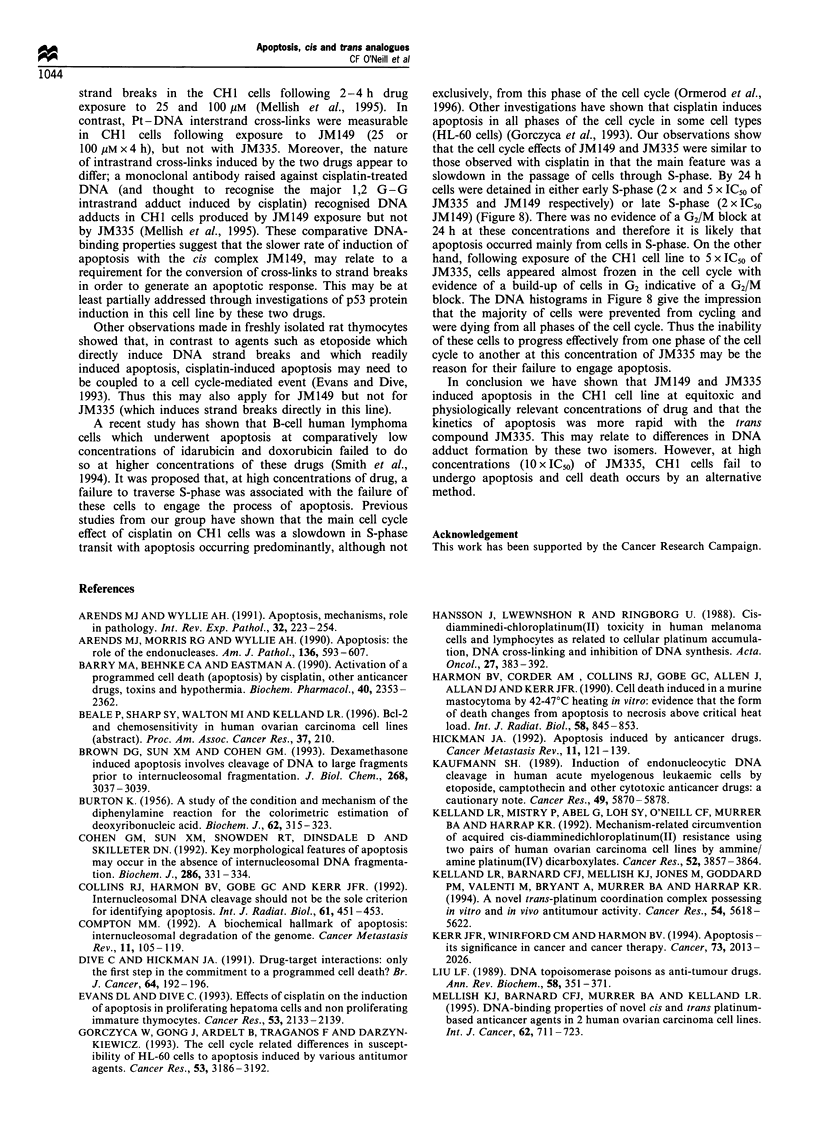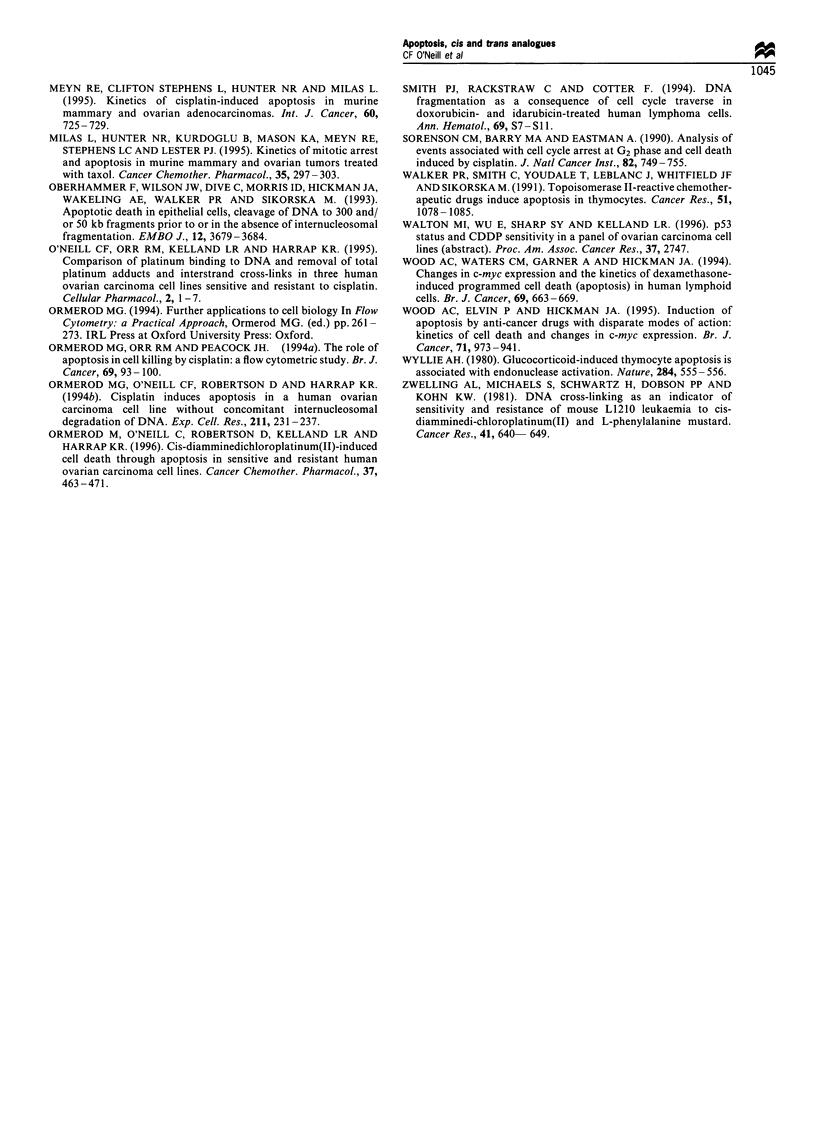# Apoptotic and non-apoptotic cell death induced by cis and trans analogues of a novel ammine(cyclohexylamine)dihydroxodichloroplatinum(IV) complex.

**DOI:** 10.1038/bjc.1996.486

**Published:** 1996-10

**Authors:** C. F. O'Neill, M. G. Ormerod, D. Robertson, J. C. Titley, Y. Cumber-Walsweer, L. R. Kelland

**Affiliations:** Cancer Research Campaign Centre for Cancer Therapeutics, Institute of Cancer Research, Sutton, Surrey, UK.

## Abstract

**Images:**


British Journal of Cancer (1996) 74, 1037-1045
© 1996 Stockton Press All rights reserved 0007-0920/96 $12.00

### Apoptotic and non-apoptotic cell death induced by *cis* and *trans* analogues of a novel ammine(cyclohexylamine)dihydroxodichloroplatinum(IV) complex

CF O'Neill, MG Ormerod, D Robertson, JC Titley, Y Cumber-Walsweer and LR Kelland

*Cancer Research Campaign Centre for Cancer Therapeutics, The Institute of Cancer Research, 15 Cotswold Road, Sutton, Surrey SM2 5NG, UK.*

**Summary** It has been previously demonstrated that cisplatin induces apoptosis in the CH1 human ovarian carcinoma cell line. This study demonstrates that two novel platinum (Pt) analogues JM149 and JM335, which are the *cis* and *trans* geometry respectively of ammine(cyclohexylamine)dihydroxodichloroPt(IV), initiate apoptosis in this cell line at physiologically relevant concentrations ($IC_{50}$ values 2 h drug exposure were 35.3 $\mu$M for JM149 and 18.7 $\mu$M for JM335). While at equimolar drug concentrations there was a 2-fold higher level of total platinum-DNA adducts following exposure to JM335 *vs* JM149, at equitoxic concentrations, levels were similar (80 *vs* 70 pmol Pt $mg^{-1}$ DNA respectively). Following a 2 h incubation with $2 \times IC_{50}$ of both drugs, cells rounded up and detached in a time-dependent manner but with the kinetics of apoptosis being more rapid for JM335. The majority of detached cells exhibited morphology associated with apoptosis which was further supported by the presence of a 50 kb fragment detected in DNA lysates prepared from these cells. JM149 induced apoptosis across a range of concentrations ($2\times$, $5\times$ and $10 \times IC_{50}$) with a 50 kb DNA fragment being detected at all concentrations. However, in marked contrast to this, JM335 failed to cause apoptosis at $10 \times IC_{50}$, the detached cells neither displaying apoptotic morphology nor a detectable 50 kb DNA fragment. Moreover, these detached cells showed evidence of extensive vesiculation while the DNA remained normal in appearance and thus appeared to have died by a non-apoptotic mode. Apoptosis also appeared to be induced to a lesser extent at $5 \times IC_{50}$ of JM335 as demonstrated by a less intense 50 kb fragment compared with that seen at $2 \times IC_{50}$. The main cell cycle effect of these drugs (at $2 \times IC_{50}$) was a slowdown in S-phase traverse during which most but not all of the apoptosis appeared to occur. However, at $5 \times IC_{50}$ of JM335 cells appeared frozen in all phases of the cell cycle with little progress from $G_1$ to S accompanied by a build-up of cells in $G_2$ indicative of a $G_2$/M block. This difference in cell cycle effect may account for the reduced level of apoptosis at this concentration and a failure to engage apoptosis at higher concentrations. These data suggest that the nature of the platinum drug (and consequently, the nature of resultant DNA damage) may have important implications in determining the rate and mechanism of cell death in this cell line. The cell death effects observed with the *trans* complex JM335 may correlate with the induction of DNA single-strand breaks in this cell line.

**Keywords:** apoptosis; *cis*/*trans* platinum analogues

Apoptosis is a biological mechanism by which cells undergo programmed cell death. Many chemotherapeutic agents of disparate mechanisms of action engage this conserved cellular response, including etoposide (Liu, 1989), camptothecin (Kaufmann, 1989), dexamethasone (Cohen *et al.*, 1992; Wood *et al.*, 1994) and taxol (Milas *et al.*, 1995). It is thought that apoptosis may be the major mechanism through which drugs mediate their cytotoxic effects (Hickman, 1992; Kerr *et al.*, 1994). Furthermore, it has been proposed that tumour sensitivity and resistance to such drugs (and thus clinical outcome) may, at least in part, be attributable to the degree of activation of a genetic programme for cell death (Dive and Hickman, 1991).

Cisplatin has been shown to induce apoptosis in a number of different cell lines including Chinese hamster ovarian cells, immature rat thymocytes, rat hepatoma cells and L1210 murine leukaemic cells (Barry *et al.*, 1990; Sorenson *et al.*, 1990; Evans and Dive, 1993; Ormerod *et al.*, 1994*a*). In recent studies, the kinetics of cisplatin-induced apoptosis have been measured *in vivo* in mammary and ovarian adenocarcinomas in rodents (Meyn *et al.*, 1995). We have demonstrated that cisplatin induced apoptosis in three human ovarian carcinoma cell lines, the CH1, the acquired resistant CH1*cis*R and the intrinsically resistant SKOV-3 without concomitant internucleosomal cleavage (Ormerod *et al.*, 1994*b*; Ormerod *et al.*, 1996). The resistance factors for CH1*cis*R and SKOV-3 following a 2 h exposure to cisplatin were 3- and 13-fold respectively (O'Neill *et al.*, 1995) with equitoxic concentrations of drug required to induce similar levels of apoptosis (Ormerod *et al.*, 1996). Furthermore, we observed that apoptosis was the major mode of cell death, occurring at concentrations ranging from the physiologically relevant $IC_{50}$ (the dose required to give 50% growth inhibition) to 10 times this dose. In the data presented here we have extended these studies to new platinum drugs. The work centres on two novel platinum compounds, JM149 and JM335, which are *cis* and *trans* analogues respectively of ammine(cyclohexylamine)dihydroxodichloroplatinum(IV). These drugs have been synthesised as part of a drug discovery programme aimed at identifying more effective platinum-based anti-cancer drugs. Recent studies have shown that JM335 exhibited a different pattern of cross-resistance from JM149 in an *in vitro* panel of sensitive and resistant cell lines (Kelland *et al.*, 1994). Furthermore, preliminary DNA binding studies in the SKOV-3 cell line demonstrated that, at equimolar concentrations, more Pt was bound to DNA with JM335 than with JM149. Moreover, JM335 like cisplatin also formed interstrand cross-links (ISC) which are thought to be the most cytotoxic of Pt/DNA lesions formed (Zwelling *et al.*, 1981; Hansson *et al.*, 1988), while JM149 did not. In contrast to this, in the CH1 cell line, while JM149 initiated very low levels of ISC formation, these were undetectable following equimolar concentrations of JM335, which instead caused DNA strand breaks (Mellish *et al.*, 1995). We have assessed

Correspondence: CF O'Neill
Received 16 February 1996; revised 19 April 1996; accepted 25 April 1996

this Pt IV *cis*/*trans* pair of Pt analogues for their ability to induce apoptosis and their effects on the cell cycle in CH1 cells.

### Materials and methods

JM149 and JM335 (Figure 1) were synthesised by and obtained from The Johnson Matthey Technology Centre (Reading, Berkshire, UK). Dulbecco's modified Eagle medium (DMEM), trypsin, phenol and cell culture supplements were purchased from Gibco/BRL (Uxbridge, Middlesex, UK). Agarose (ultra pure), fluorescein diacetate (FDA), propidium iodide(PI), pulse field molecular weight markers and all other reagent chemicals were purchased from Sigma Chemical Co. Ltd. (Poole, Dorset, UK).

### *Cell culture and cytotoxicity*

The CH1 human ovarian carcinoma was cultured as a monolayer in DMEM supplemented with 10% heat-inactivated fetal calf serum (FCS), MEM non-essential amino acids, 2 mM glutamine, 5 $\mu g\ ml^{-1}$ insulin, 0.4 $\mu g\ ml^{-1}$ hydrocortisone, 2.5 $\mu g\ ml^{-1}$ amphotericin and 50 ng $ml^{-1}$ gentamicin. JM149 and JM335 were dissolved in sterile 0.9% sodium chloride solution and $IC_{50}$ values determined at 96 h following a 2 h exposure to drug by the sulphorhodamine B assay (SRB) as previously described (Kelland *et al.*, 1992). The rate of cellular detachment was measured over a 72 h period following a 2 h incubation with 2 × and 5 × the $IC_{50}$ of both drugs.

### *Measurement of platinum bound to DNA*

Following a 2 h incubation with equimolar concentrations of JM149 and JM335, DNA was extracted using the phenol method as previously described (O'Neill *et al.*, 1995). Dried DNA pellets were dissolved overnight at 37°C in 0.2% nitric acid and platinum content measured by flameless atomic absorption spectroscopy (FAAS) using a Perkin Elmer 1100B/HGA 700. DNA content was measured using the Burton assay, a colorimetric method which quantitates 2′-deoxyribose units (Burton, 1956). Results were expressed as pmols of platinum (Pt) per mg of DNA. The DNA extraction efficiency was an average of 90 $\mu g \pm 10$ per $10^7$ cells.

### *Microscopy*

Cells were exposed to 2 × and 10 × $IC_{50}$ concentrations of JM149 and JM335 for 2 h and collected 24 h later. Detached cells were harvested from the culture medium by centrifugation with attached cells being removed by incubation with 0.2% trypsin for 5 min before collection. Cell pellets were washed in phosphate-buffered saline (PBS) and fixed in 2% glutaraldehyde in 0.05 M PBS, 0.05 M sucrose, pH 7.3, for 2 h at room temperature. Following this, pellets were post-fixed in 1% osmium tetroxide, dehydrated through a graded series of ethanols, infiltrated and embedded in epon. For light microscopy, 1 $\mu m$ sections were cut and stained with toluidine blue. For electron microscopy, 0.1 $\mu m$ sections were picked up on copper grids and double stained with uranyl acetate and lead citrate and viewed with a Philips CM10 using 80 kv accelerating voltage.

### *Flow cytometry*

For cell cycle analysis, cultures were exposed to 2 × and 5 × $IC_{50}$ concentrations of JM149 and JM335 for 2 h and attached cells washed, incubated with trypsin as above and collected by centrifugation at 0, 2, 5, 16 and 24 h following removal of drug. Cell pellets were resuspended in 200 $\mu l$ ice-cold PBS and fixed for at least 30 min in 1.8 ml ice-cold 70% ethanol. The fixed cells were centrifuged and the pellets resuspended in 800 $\mu l$ PBS, 100 $\mu l$ (1 mg $ml^{-1}$) RNAase and 100 $\mu l$ of PI (200 $\mu g\ ml^{-1}$) and left to incubate at 37°C for 30 min. Flow cytometry was carried out on a Coulter Elite equipped with a Spectra-physics argon-ion laser with an output of 200 mW at 488 nm. Data from $2 \times 10^4$ cells were analysed with forward and orthogonally scattered light and red fluorescence (peak and integrated area) recorded. Pulse shape analysis was performed to eliminate any cell clumps and data were gated on light scatter before recording a histogram of red (PI-DNA) fluorescence (Ormerod *et al.*, 1994*a*).

### *Viability studies*

Cell viability was estimated by flow cytometry using the fluorescein diacetate/propidium iodide (FDA/PI) method (Ormerod, 1994); the cells were incubated in medium at room temperature for 10 min with 50 ng $ml^{-1}$ FDA. PI at 5 $\mu g\ ml^{-1}$ was added and green and red fluorescence recorded in a flow cytometer using an argon-ion laser tuned to 488 nm. Green-positive, red-negative cells were scored as viable, green-negative, red-positive cells as dead.

### *DNA gel electrophoresis*

Cells were incubated with 2 ×, 5 × and 10 × $IC_{50}$ of JM149 and JM335 with attached and detached cells collected 24 h later by centrifugation. Cell pellets (approximately $5 \times 10^5$ per 50 $\mu l$) were incubated for 1 h at 37°C in a lysis buffer [200 mM Tris, 100 mM EDTA, 2% sodium dodecyl sulphate (SDS)] containing 1 mg $ml^{-1}$ proteinase K final concentration. An aliquot of 10 $\mu l$ of a 1 mg $ml^{-1}$ solution of RNAase per 50 $\mu l$ of sample was added and incubation continued for a further hour. Aliquots of cell lysate were added directly to the gel, the wells being sealed with 1% low melting point agarose. Field inversion gel electrophoresis (FIGE) was performed with 1 × TAE (40 mM Tris, 20 mM sodium acetate and 1 mM EDTA) using a Bio Rad FIGE Mapper. Horizontal gels were run for 20 h in 1 × TAE buffer with a forward voltage of 10 V $cm^{-1}$ and reverse of 7 V $cm^{-1}$ with linear ramping $T_1 = 1$ s to $T_2 = 12$ s. The temperature of the TAE buffer was controlled to 14°C using a Bio Rad 1000 mini-chiller. Sigma Pulse Marker $\lambda$ fragments 0.1–200 kb and *S. cerevisiae* fragments 225–2200 kb were run with the samples.

**Figure 1** The structures of JM149 and JM335.

### Results

### *Cytotoxicity*

The cytotoxicity of both drugs was measured using the SRB assay 96 h following a 2 h exposure to drug (Figure 2). The $IC_{50}$ s were 35.3±1.5 $\mu$M for JM149 and 18.7±8.4 $\mu$M for JM335; this difference just attained statistical significance ($P=0.03$). This gave an approximately 1.9-fold difference in activity, similar to that seen in previous studies following continuous exposure to drug for 96 h (Kelland *et al.*, 1994).

### *Platinum bound to DNA*

The amount of Pt bound to DNA was measured following a 2 h incubation with 25, 50 and 100 $\mu$M JM149 and JM335 (Figure 3). There was approximately twice as much Pt bound to DNA following incubation with JM335 compared with JM149. For example, incubation with 50 $\mu$M drug resulted in 50±2 pmol $mg^{-1}$ DNA for JM149 and 110.5 ±35.5 pmol $mg^{-1}$ DNA for JM335 ($P$-value 0.04). However, at equitoxic concentrations ($2\times IC_{50}$) total platinum levels on DNA (i.e. inclusive of monofunctional and bifunctional adducts) were similar for the two isomers; JM149 at 70 $\mu$M, 70 pmol $mg^{-1}$ DNA, JM335 at 37 $\mu$M, 80 pmol $mg^{-1}$ DNA.

### *Cell death induced by JM149 and JM335*

The detachment of cells was measured after a 2 h incubation with 2× and $5\times IC_{50}$ of JM149 and JM335, both attached and detached cells being counted at 0, 5, 16, 24, 48, and 72 h following the removal of drug. The number of attached cells remaining at each time point was calculated as a percentage of cell number at time 0 (Figure 4a). Following exposure to JM149 numbers of attached cells continued to increase for up to 24 h and 16 h after 2× and 5× the $IC_{50}$ respectively, while no increase in the number of the attached cell population was observed after incubation with either concentration of JM335.

The number of detached cells was calculated as a percentage of the total number of cells present at each time point so as to take into account any increase in cell number following removal of drug (Figure 4b). At equitoxic concentrations of drug, JM335 initiated cellular detachment at a much faster rate than JM149 which appeared to exhibit a time lag for up to 15 h following removal of drug. By 24 h following removal of drug, approximately 77% and 82%

**Figure 2** Cytotoxicity of JM149 (■) and JM335 (▲) in the CH1 cell line following 2 h drug exposure. Error bars represent s.d. where $n=3$.

**Figure 3** Platinum binding to DNA following a 2 h exposure to 25, 50 and 100 $\mu$M JM149 (■) and JM335 (▲). Error bars represent s.d. where $n=3$.

**Figure 4** (**a**) Attached cells remaining as a percentage of cell number at time 0 and (**b**) detached cells as a percentage of total cell number at each time point following a 2 h exposure to 2× (■) and 5× (▲) the $IC_{50}$ of JM149 and 2×(◆) and 5× (●) the $IC_{50}$ of JM335. Error bars represent s.d. where $n=3$.

**Figure 5** Light microscopy of CH1 cells harvested 24 h following exposure to 2 × and 10 × the $IC_{50}$ of drug. (**a**) Control untreated attached cells. (**b**) Attached cells at 2 × JM149. (**c**) Attached cells at 2 × JM335. (**d** and **e**) Detached cells at 2 × JM149 and JM335. (**f** and **g**) Detached cells at 10 × JM149 and JM335. Original magnification × 600, bar = 25 μm.

respectively of cells had detached following $2\times$ and $5\times IC_{50}$ JM335 compared with only 17% and 43% for JM149. The majority of JM149-treated cells had detached by 72 h. The combined total of attached and detached cells 24 h after exposure to either drug at either concentration was similar [e.g. JM149 ($2\times IC_{50}$) $2.8\times10^6$ cells, ($5\times IC_{50}$) $2.3\times10^6$ cells; JM335 ($2\times IC_{50}$) $2.4\times10^6$ cells, ($5\times IC_{50}$) $2.6\times10^6$ cells]. Thus, although cell numbers increased with JM335, these cells appeared to be dying and detaching immediately following cell division whereas the majority of the JM149-treated cells remained attached.

*Morphology and viability of attached and detached cells*

The morphology of attached and detached cells was examined by light and electron microscopy 24 h following a 2 h incubation with $2\times$ and $10\times IC_{50}$ of JM149 and JM335. The attached cells remaining after incubation with $2\times IC_{50}$ of JM149 showed no evidence of chromatin condensation associated with apoptosis, with the chromatin remaining similar in appearance to that of control untreated cells (Figure 5a and b). On the other hand while the majority of the attached cells remaining after $2\times IC_{50}$ of JM335 exhibited normal morphology, there was evidence that some apoptosis had taken place in that there were a small number of cells showing evidence of chromatin condensation (arrowed on Figure 5c). At $2\times IC_{50}$ of both drugs the majority of detached cells exhibited the morphology consistent with apoptosis, displaying the characterisatic condensation and fragmentation pattern of chromatin around the periphery of the nucleus (Figure 5d and e). At $10\times IC_{50}$ of JM149, the detached cells were again apoptotic in appearance (Figure 5f and Figure 6a), however, in contrast to this, the detached cells induced by the *trans* complex, JM335, at this concentration did not exhibit morphology associated with apoptosis (Figure 5g and Figure 6b). The chromatin was not condensed and resembled that of the attached cells. Moreover, there was evidence of extensive vesiculation in these cells which could be seen more clearly upon closer examination by electron microscopy (Figure 6b). Cell viability was estimated by measuring the number of cells with intact plasma membranes by flow cytometry 24 h following a 2 h incubation with both drugs. Across the range of concentrations of both drugs the average percentage of viable cells was 91% for the attached cells and between 35% and 64% for the detached cells (Table I).

*DNA gel electrophoresis*

FIGE was carried out on cell lysates prepared from attached and detached cells harvested 24 h after a 2 h exposure to $2\times$, $5\times$ and $10\times IC_{50}$ of JM149 and JM335. A fragment approximately 50 kb in size, was detected in the detached cells obtained with $2\times$, $5\times$ and $10\times IC_{50}$ of JM149. This fragment was readily observable in the detached cells following $2\times IC_{50}$ of JM335, was less apparent at $5\times IC_{50}$ but was not detectable at $10\times IC_{50}$ JM335 (Figure 7). This fragment was also absent from the attached cells at all concentrations of both drugs. Furthermore, internucleosomal cleavage could not be detected in the cells undergoing apoptosis (data not shown).

*Cell cycle analysis*

Flow cytometric analysis of the effects of $2\times$ and $5\times IC_{50}$ of JM149 and JM335 on the progression of cells through the cell cycle was carried out on attached cells only. The DNA histograms indicated that the main effect of both concentrations of JM149 and $2\times IC_{50}$ of JM335 was a slowdown of passage through S-phase (Figure 8) as evidenced by the increase in the percentage of cells in this phase of the cell cycle by 16 h and 24 h following removal of drug (Figure 9). By 24 h, cells exposed to $2\times IC_{50}$ of JM335 and $5\times IC_{50}$ of JM149 had not progressed beyond early S-phase, while those exposed to $2\times IC_{50}$ JM149 had progressed to late S-phase (Figure 8). However, at $5\times IC_{50}$ of JM335 cells seemed almost frozen in the cell cycle with little movement from $G_1$ into S and this was accompanied by a build-up of cells in $G_2$ representative of a $G_2/M$ block (Figure 9).

**Figure 6** Electron micrographs of detached cells. (**a**) $10\times$ JM149 and (**b**) $10\times$ JM335. Original magnification: **a** $\times 5000$; **b** $\times 9000$. (**a**) bar 2 $\mu$m; (**b**) bar 4 $\mu$m.

**Table I** Percentage viability of attached and detached cells measured 24 h following a 2 h incubation with JM149 and JM335

| | $IC_{50}$ | *Percentage viability of attached cells* | *Percentage viability of detached cells* |
|---|---|---|---|
| JM149 | ×2 | 99±0.0 | 41.2±1.4 |
| | ×5 | 88.5±10.4 | 43.3±6.2 |
| | ×10 | 84.8±18.4 | 39.6±11.8 |
| JM335 | ×2 | 91.4±6.2 | 35.2±4.7 |
| | ×5 | 94.5±1.7 | 41.6±26 |
| | ×10 | 90.1±3.8 | 64±2.1 |

### Discussion

We have demonstrated the potential of two novel isomeric platinum analogues JM149 (*cis*) and JM335 (*trans*) ammine (cyclohexylamine)dihydroxodichloroplatinum (IV) (Kelland *et al.*, 1994) to induce apoptosis in the CH1 human ovarian carcinoma cell line. This cell line has previously been shown to be relatively sensitive to cisplatin (e.g. Kelland *et al.*, 1994) and to undergo apoptosis following exposure to physiologically relevant concentrations of cisplatin (Ormerod *et al.*, 1994*b*, 1996). Following a 2 h exposure to various concentrations of cisplatin, cells rounded up and detached from the monolayer in a time- and dose-dependent manner with the majority of cells detaching at between 24 and 48 h following removal of drug. Electron and light microscopy revealed that the detached cells from both CH1 cell lines displayed typical morphological features of apoptosis with the DNA degraded into fragments of 30-50 kb in size. The major cell cycle effect appeared to be S-phase slowdown with a comparatively small $G_2$ block. Studies have shown that CH1 cells possess a wild-type p53 gene sequence, which is induced approximately 5-fold following 5 Gy gamma-irradiation (Walton *et al.*, 1996) and express Bcl-2 protein (Beale *et al.*, 1996).

**Figure 7** FIGE of attached and detached cells collected 24 h following a 2 h exposure to $2\times$, $5\times$ and $10\times IC_{50}$ of JM149 and JM335. Lanes: 1, *S. cerevisae* standards 225–2200 kb; 2 and 15, $\lambda$ fragments 0.1–200 kb; 3–5, JM149 attached cells; 6–8, JM335 attached cells; 9–11, JM149 detached cells; 12–14, JM335 detached cells.

Both platinum-based isomers induced apoptosis in this cell line at $2\times IC_{50}$, a concentration which is roughly equivalent to a 95% inhibition of cell growth. The detached cells elicited by the two drugs exhibited fragmentation and condensation of chromatin around the periphery of the nuclear membrane (Figure 5d and e) consistent with the morphology of apoptosis (Wyllie, 1980; Arends *et al.*, 1990; Arends and Wyllie 1991). That apoptosis had indeed occurred was further substantiated by the presence of a 50 kb DNA fragment (Walker *et al.*, 1991; Oberhammer *et al.*, 1993; Brown *et al.*, 1993; Ormerod *et al.*, 1994*b* and 1996) observed in the

**Figure 8** DNA histograms showing the cell cycle changes with time (h) following a 2 h exposure to $2\times$ and $5\times IC_{50}$ of JM149 and JM335.

**Figure 9** Percentage number of cells in each phase of the cell cycle measured over a 24 h period following a 2 exposure to JM149 and JM335. Open symbols, $2\times IC_{50}$ and closed symbols, $5\times IC_{50}$ ($G_1$ □, ■) (S △, ▲) ($G_2$ ◇, ◆).

detached cells induced by both drugs on FIGE (Figure 7). In many cases the DNA of apoptotic cells is ultimately cleaved at internucleosomal sites into fragments of 180 bp and multiples thereof (Arends *et al.*, 1990; Compton, 1992) giving a DNA ladder effect on gel electrophoresis. In common with our studies with cisplatin, internucleosomal degradation was not observed in the apoptotic cells induced by either JM149 or JM335 in the CH1 cell line. There was no morphological evidence of apoptosis in the attached cells remaining following JM149 (Figure 5b), but a small number of apoptotic-like cells were identified in the attached cell population following exposure to JM335 (Figure 5c). However, none of the attached cells exhibited the presence of a 50 kb fragment on FIGE, not even at the highest concentrations of both drugs. Thus the level of apoptosis seen in the attached cells following JM335 was insufficient under these experimental conditions to yield a detectable 50 kb fragment. Interestingly, cell viability studies showed that around 40% of the detached cells retained intact plasma membranes at 24 h which is comparatively lower than that seen with cisplatin, in which over 60% of detached apoptotic cells maintained plasma membrane integrity (Ormerod *et al.*, 1994*b*).

The *cis* analogue JM149 induced apoptosis across the wide range of concentrations used ($2\times$, $5\times$ and $10\times IC_{50}$) as evidenced by either change in morphology or the presence of a 50 kb fragment, with features of apoptosis being observed only in the detached cell population. In striking contrast to this, the *trans* isomer JM335 did not induce apoptosis at $10\times IC_{50}$. The chromatin of the detached cells remained normal in appearance with no morphological evidence of apoptosis having taken place (Figure 5g). Moreover, no 50 kb DNA fragment or fragments of a similar size were detected on FIGE (Figure 7). Notably, the detached cells displayed extensive vesiculation of the cytoplasm at this concentration which was not observed in cells undergoing apoptosis following the lower dose of this drug. The reason for this is unclear but may have been due to loss of osmotic control and subsequent water imbalance. In certain respects the vesiculation observed with JM335 resembles structures observed in cells undergoing necrosis following heating to temperatures of between 42 and 47°C (Harmon *et al.*, 1990), but in the case of JM335, vesiculation occurred on a greater scale occupying in some cases almost 50% of cell volume. Furthermore, very few of these cells displayed the irregular fragmentation pattern of condensed chromatin associated with cells undergoing necrosis (Harmon *et al.*, 1990; Collins *et al.*, 1992) and intriguingly a higher proportion of these detached cells (64%) had intact cell membranes at 24 h compared with the apoptotic cells. This does not, however, eliminate necrosis as the mode of cell death in these cells.

It can be seen in the detached cell population that while there was a strong 50 kb band at $2\times IC_{50}$ of JM335, this was less intense at $5\times IC_{50}$ and undetectable at $10\times IC_{50}$. Great care was exercised to ensure that equal numbers of cells were loaded into each well in order to reduce variance in nuclear material between samples; thus it can be reasonably assumed that the difference in band intensity seen between $2\times$ and $5\times IC_{50}$ of this drug was entirely due to a smaller number of cells undergoing apoptosis at the higher concentration. We conclude, therefore, that the complete absence of 50 kb fragment and lack of apoptotic morphology indicates that cell death did not occur through apoptosis at $10\times IC_{50}$ of JM335, with cells dying by an alternative process.

Another striking difference in activity between the two drugs was that at both $2\times$ and $5\times IC_{50}$ of JM149, cells appear to have experienced a lag phase (approximately 15 h) to the onset of apoptosis, whereas at $2\times IC_{50}$ of JM335 the rate of induction of apoptosis is almost linear (Figure 4b). Moreover, while at $2\times IC_{50}$ of JM149 there was a measurable increase in the number of attached cells up to 24 h, no increase in the attached cell population was observed with either concentration of JM335 (Figure 4a). However, at 24 h after drug incubation the combined totals for attached and detached cells were similar for each concentration of both JM149 and JM335. Thus it is possible that following exposure to JM335, cells underwent aberrant division and the dividing cells detached immediately thereafter. Other studies have shown that drugs of disparate structures and mechanisms of action display a differential time lag to the induction of apoptosis and that there are differences in the way various agents ultimately induce apoptosis (Wood *et al.*, 1995). However, JM149 and JM335 differ only in the orientation of a chlorine and ammine group around a central Pt. Nonetheless, JM335 induced apoptosis at a much greater rate than JM149, such that at $2\times IC_{50}$ of both drugs over four times as many cells had undergone apoptosis by 24 h with the *trans* compound. In addition, this concentration of JM335 induced apoptosis at a faster rate than $5\times IC_{50}$ of JM149.

Interestingly, the more rapid kinetics of apoptosis seen with JM335 does not appear to be related to the amount of total Pt binding to DNA. While at equimolar concentrations this is 2-fold higher for JM335 compared with JM149 (approximately 110 and 50 pmol $mg^{-1}$ DNA respectively at 50 $\mu$M), at equitoxic concentrations of both drugs the levels of Pt bound to DNA are similar (80 *vs* 70 pmol Pt $mg^{-1}$ DNA at $2\times IC_{50}$). Thus the more rapid induction of apoptosis by JM335 may relate to differences in the nature of DNA damage induced by the two isomers in this cell line. Notably, JM335, but not JM149, was shown to induce DNA

strand breaks in the CH1 cells following 2–4 h drug exposure to 25 and 100 $\mu$M (Mellish *et al.*, 1995). In contrast, Pt–DNA interstrand cross-links were measurable in CH1 cells following exposure to JM149 (25 or 100 $\mu M \times 4$ h), but not with JM335. Moreover, the nature of intrastrand cross-links induced by the two drugs appear to differ; a monoclonal antibody raised against cisplatin-treated DNA (and thought to recognise the major 1,2 G–G intrastrand adduct induced by cisplatin) recognised DNA adducts in CH1 cells produced by JM149 exposure but not by JM335 (Mellish *et al.*, 1995). These comparative DNA-binding properties suggest that the slower rate of induction of apoptosis with the *cis* complex JM149, may relate to a requirement for the conversion of cross-links to strand breaks in order to generate an apoptotic response. This may be at least partially addressed through investigations of p53 protein induction in this cell line by these two drugs.

Other observations made in freshly isolated rat thymocytes showed that, in contrast to agents such as etoposide which directly induce DNA strand breaks and which readily induced apoptosis, cisplatin-induced apoptosis may need to be coupled to a cell cycle-mediated event (Evans and Dive, 1993). Thus this may also apply for JM149 but not for JM335 (which induces strand breaks directly in this line).

A recent study has shown that B-cell human lymphoma cells which underwent apoptosis at comparatively low concentrations of idarubicin and doxorubicin failed to do so at higher concentrations of these drugs (Smith *et al.*, 1994). It was proposed that, at high concentrations of drug, a failure to traverse S-phase was associated with the failure of these cells to engage the process of apoptosis. Previous studies from our group have shown that the main cell cycle effect of cisplatin on CH1 cells was a slowdown in S-phase transit with apoptosis occurring predominantly, although not exclusively, from this phase of the cell cycle (Ormerod *et al.*, 1996). Other investigations have shown that cisplatin induces apoptosis in all phases of the cell cycle in some cell types (HL-60 cells) (Gorczyca *et al.*, 1993). Our observations show that the cell cycle effects of JM149 and JM335 were similar to those observed with cisplatin in that the main feature was a slowdown in the passage of cells through S-phase. By 24 h cells were detained in either early S-phase ($2\times$ and $5\times IC_{50}$ of JM335 and JM149 respectively) or late S-phase ($2\times IC_{50}$ JM149) (Figure 8). There was no evidence of a $G_2$/M block at 24 h at these concentrations and therefore it is likely that apoptosis occurred mainly from cells in S-phase. On the other hand, following exposure of the CH1 cell line to $5\times IC_{50}$ of JM335, cells appeared almost frozen in the cell cycle with evidence of a build-up of cells in $G_2$ indicative of a $G_2$/M block. The DNA histograms in Figure 8 give the impression that the majority of cells were prevented from cycling and were dying from all phases of the cell cycle. Thus the inability of these cells to progress effectively from one phase of the cell cycle to another at this concentration of JM335 may be the reason for their failure to engage apoptosis.

In conclusion we have shown that JM149 and JM335 induced apoptosis in the CH1 cell line at equitoxic and physiologically relevant concentrations of drug and that the kinetics of apoptosis was more rapid with the *trans* compound JM335. This may relate to differences in DNA adduct formation by these two isomers. However, at high concentrations ($10\times IC_{50}$) of JM335, CH1 cells fail to undergo apoptosis and cell death occurs by an alternative method.

**Acknowledgement**
This work has been supported by the Cancer Research Campaign.

### References

ARENDS MJ AND WYLLIE AH. (1991). Apoptosis, mechanisms, role in pathology. *Int. Rev. Exp. Pathol.*, **32,** 223–254.
ARENDS MJ, MORRIS RG AND WYLLIE AH. (1990). Apoptosis: the role of the endonucleases. *Am. J. Pathol.*, **136,** 593–607.
BARRY MA, BEHNKE CA AND EASTMAN A. (1990). Activation of a programmed cell death (apoptosis) by cisplatin, other anticancer drugs, toxins and hypothermia. *Biochem. Pharmacol.*, **40,** 2353–2362.
BEALE P, SHARP SY, WALTON MI AND KELLAND LR. (1996). Bcl-2 and chemosensitivity in human ovarian carcinoma cell lines (abstract). *Proc. Am. Assoc. Cancer Res.*, **37,** 210.
BROWN DG, SUN XM AND COHEN GM. (1993). Dexamethasone induced apoptosis involves cleavage of DNA to large fragments prior to internucleosomal fragmentation. *J. Biol. Chem.*, **268,** 3037–3039.
BURTON K. (1956). A study of the condition and mechanism of the diphenylamine reaction for the colorimetric estimation of deoxyribonucleic acid. *Biochem. J.*, **62,** 315–323.
COHEN GM, SUN XM, SNOWDEN RT, DINSDALE D AND SKILLETER DN. (1992). Key morphological features of apoptosis may occur in the absence of internucleosomal DNA fragmentation. *Biochem. J.*, **286,** 331–334.
COLLINS RJ, HARMON BV, GOBE GC AND KERR JFR. (1992). Internucleosomal DNA cleavage should not be the sole criterion for identifying apoptosis. *Int. J. Radiat. Biol.*, **61,** 451–453.
COMPTON MM. (1992). A biochemical hallmark of apoptosis: internucleosomal degradation of the genome. *Cancer Metastasis Rev.*, **11,** 105–119.
DIVE C AND HICKMAN JA. (1991). Drug-target interactions: only the first step in the commitment to a programmed cell death? *Br. J. Cancer*, **64,** 192–196.
EVANS DL AND DIVE C. (1993). Effects of cisplatin on the induction of apoptosis in proliferating hepatoma cells and non proliferating immature thymocytes. *Cancer Res.*, **53,** 2133–2139.
GORCZYCA W, GONG J, ARDELT B, TRAGANOS F AND DARZYNKIEWICZ. (1993). The cell cycle related differences in susceptibility of HL-60 cells to apoptosis induced by various antitumor agents. *Cancer Res.*, **53,** 3186–3192.
HANSSON J, LWEWNSHON R AND RINGBORG U. (1988). Cis-diamminedi-chloroplatinum(II) toxicity in human melanoma cells and lymphocytes as related to cellular platinum accumulation, DNA cross-linking and inhibition of DNA synthesis. *Acta. Oncol.*, **27,** 383–392.
HARMON BV, CORDER AM , COLLINS RJ, GOBE GC, ALLEN J, ALLAN DJ AND KERR JFR. (1990). Cell death induced in a murine mastocytoma by 42-47°C heating *in vitro*: evidence that the form of death changes from apoptosis to necrosis above critical heat load. *Int. J. Radiat. Biol.*, **58,** 845–853.
HICKMAN JA. (1992). Apoptosis induced by anticancer drugs. *Cancer Metastasis Rev.*, **11,** 121–139.
KAUFMANN SH. (1989). Induction of endonucleocytic DNA cleavage in human acute myelogenous leukaemic cells by etoposide, camptothecin and other cytotoxic anticancer drugs: a cautionary note. *Cancer Res.*, **49,** 5870–5878.
KELLAND LR, MISTRY P, ABEL G, LOH SY, O'NEILL CF, MURRER BA AND HARRAP KR. (1992). Mechanism-related circumvention of acquired cis-diamminedichloroplatinum(II) resistance using two pairs of human ovarian carcinoma cell lines by ammine/amine platinum(IV) dicarboxylates. *Cancer Res.*, **52,** 3857–3864.
KELLAND LR, BARNARD CFJ, MELLISH KJ, JONES M, GODDARD PM, VALENTI M, BRYANT A, MURRER BA AND HARRAP KR. (1994). A novel *trans*-platinum coordination complex possessing *in vitro* and *in vivo* antitumour activity. *Cancer Res.*, **54,** 5618–5622.
KERR JFR, WINIRFORD CM AND HARMON BV. (1994). Apoptosis – its significance in cancer and cancer therapy. *Cancer*, **73,** 2013–2026.
LIU LF. (1989). DNA topoisomerase poisons as anti-tumour drugs. *Ann. Rev. Biochem.*, **58,** 351–371.
MELLISH KJ, BARNARD CFJ, MURRER BA AND KELLAND LR. (1995). DNA-binding properties of novel *cis* and *trans* platinum-based anticancer agents in 2 human ovarian carcinoma cell lines. *Int. J. Cancer*, **62,** 711–723.

MEYN RE, CLIFTON STEPHENS L, HUNTER NR AND MILAS L. (1995). Kinetics of cisplatin-induced apoptosis in murine mammary and ovarian adenocarcinomas. *Int. J. Cancer*, **60**, 725–729.

MILAS L, HUNTER NR, KURDOGLU B, MASON KA, MEYN RE, STEPHENS LC AND LESTER PJ. (1995). Kinetics of mitotic arrest and apoptosis in murine mammary and ovarian tumors treated with taxol. *Cancer Chemother. Pharmacol.*, **35**, 297–303.

OBERHAMMER F, WILSON JW, DIVE C, MORRIS ID, HICKMAN JA, WAKELING AE, WALKER PR AND SIKORSKA M. (1993). Apoptotic death in epithelial cells, cleavage of DNA to 300 and/or 50 kb fragments prior to or in the absence of internucleosomal fragmentation. *EMBO J.*, **12**, 3679–3684.

O'NEILL CF, ORR RM, KELLAND LR AND HARRAP KR. (1995). Comparison of platinum binding to DNA and removal of total platinum adducts and interstrand cross-links in three human ovarian carcinoma cell lines sensitive and resistant to cisplatin. *Cellular Pharmacol.*, **2**, 1–7.

ORMEROD MG. (1994). Further applications to cell biology In *Flow Cytometry: a Practical Approach*, Ormerod MG. (ed.) pp. 261–273. IRL Press at Oxford University Press: Oxford.

ORMEROD MG, ORR RM AND PEACOCK JH. (1994*a*). The role of apoptosis in cell killing by cisplatin: a flow cytometric study. *Br. J. Cancer*, **69**, 93–100.

ORMEROD MG, O'NEILL CF, ROBERTSON D AND HARRAP KR. (1994*b*). Cisplatin induces apoptosis in a human ovarian carcinoma cell line without concomitant internucleosomal degradation of DNA. *Exp. Cell. Res.*, **211**, 231–237.

ORMEROD M, O'NEILL C, ROBERTSON D, KELLAND LR AND HARRAP KR. (1996). Cis-diamminedichloroplatinum(II)-induced cell death through apoptosis in sensitive and resistant human ovarian carcinoma cell lines. *Cancer Chemother. Pharmacol.*, **37**, 463–471.

SMITH PJ, RACKSTRAW C AND COTTER F. (1994). DNA fragmentation as a consequence of cell cycle traverse in doxorubicin- and idarubicin-treated human lymphoma cells. *Ann. Hematol.*, **69**, S7–S11.

SORENSON CM, BARRY MA AND EASTMAN A. (1990). Analysis of events associated with cell cycle arrest at $G_2$ phase and cell death induced by cisplatin. *J. Natl Cancer Inst.*, **82**, 749–755.

WALKER PR, SMITH C, YOUDALE T, LEBLANC J, WHITFIELD JF AND SIKORSKA M. (1991). Topoisomerase II-reactive chemotherapeutic drugs induce apoptosis in thymocytes. *Cancer Res.*, **51**, 1078–1085.

WALTON MI, WU E, SHARP SY AND KELLAND LR. (1996). p53 status and CDDP sensitivity in a panel of ovarian carcinoma cell lines (abstract). *Proc. Am. Assoc. Cancer Res.*, **37**, 2747.

WOOD AC, WATERS CM, GARNER A AND HICKMAN JA. (1994). Changes in c-*myc* expression and the kinetics of dexamethasone-induced programmed cell death (apoptosis) in human lymphoid cells. *Br. J. Cancer*, **69**, 663–669.

WOOD AC, ELVIN P AND HICKMAN JA. (1995). Induction of apoptosis by anti-cancer drugs with disparate modes of action: kinetics of cell death and changes in c-*myc* expression. *Br. J. Cancer*, **71**, 973–941.

WYLLIE AH. (1980). Glucocorticoid-induced thymocyte apoptosis is associated with endonuclease activation. *Nature*, **284**, 555–556.

ZWELLING AL, MICHAELS S, SCHWARTZ H, DOBSON PP AND KOHN KW. (1981). DNA cross-linking as an indicator of sensitivity and resistance of mouse L1210 leukaemia to cis-diamminedi-chloroplatinum(II) and L-phenylalanine mustard. *Cancer Res.*, **41**, 640— 649.